# Correction: Chen et al. *VdPT1* Encoding a Neutral Trehalase of *Verticillium dahliae* Is Required for Growth and Virulence of the Pathogen. *Int. J. Mol. Sci.* 2024, *25*, 294

**DOI:** 10.3390/ijms27115110

**Published:** 2026-06-05

**Authors:** Lihua Chen, Xiaohu Ma, Tiange Sun, Qian-Hao Zhu, Hongjie Feng, Yongtai Li, Feng Liu, Xinyu Zhang, Jie Sun, Yanjun Li

**Affiliations:** 1 The Key Laboratory of Oasis Eco-Agriculture, Agriculture College, Shihezi University, Shihezi 832000, China; chenlihua@stu.shzu.edu.cn (L.C.); xhma@stu.shzu.edu.cn (X.M.); suntiange@stu.shzu.edu.cn (T.S.); liyongtai@stu.shzu.edu.cn (Y.L.); liufeng@shzu.edu.cn (F.L.); zhxy@shzu.edu.cn (X.Z.); 2 CSIRO Agriculture and Food, GPO Box 1700, Canberra 2601, Australia; Qianhao.Zhu@csiro.au; 3 State Key Laboratory of Cotton Biology, Institute of Cotton Research of Chinese Academy of Agricultural Sciences, Anyang 455000, China; fenghongjie@caas.cn

In the original publication [1], there were mistakes in Figure 7A (*ΔVdPT1-1*/18dpi, *ΔVdPT1-2*/18dpi, *ΔVdPT1-C*/14dpi), Figure 8A (Mock/21dpi, pTRV2-*00*/14dpi, pTRV2-*VdPT1*/21dpi) and Figure 8B (pTRV2-*00*/14dpi, pTRV2-*VdPT1*/14dpi). The aforementioned images need to be corrected. The corrected Figure 7 and Figure 8 appear below. The authors state that the scientific conclusions are unaffected. This correction was approved by the Academic Editor. The original publication has also been updated.

## Figures and Tables

**Figure 7:**
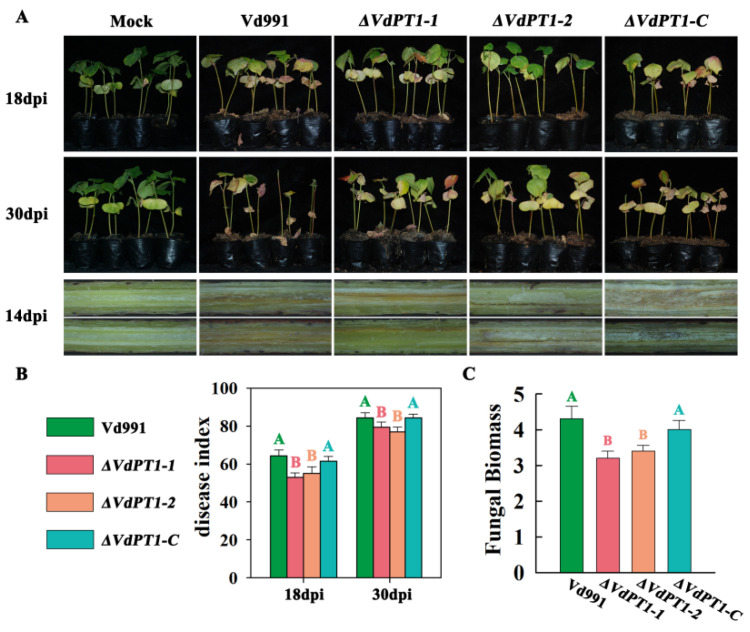
Comparison of pathogenicity of different *V. dahliae* strains. (**A**) Disease symptoms of cotton plants infected with different strains at 18 dpi and 30 dpi. Vascular browning observation was performed at 14 dpi. (**B**) Disease index of cotton plants infected with different strains at 18 dpi and 30 dpi. (**C**) qRT-PCR assay of fungal biomass in cotton plants treated with different strains at 21 dpi. Data were statistically analyzed using IBM SPSS statistics 26.0. Significant differences between different treatments were analyzed using Duncan’s multiple-range tests. Different letters above the error bars indicate statistically significant differences at *p* < 0.01 from one-way ANOVA tests.

**Figure 8:**
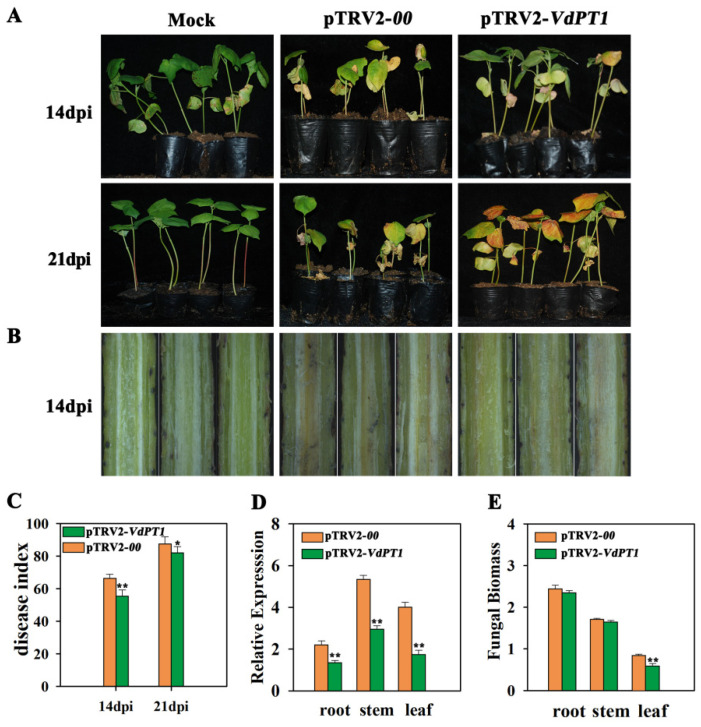
Functional assessment of *VdPT1* in the pathogenicity of *V. dahliae* by TRV-based HIGS. (**A**) Fungal infection symptoms of cotton seedlings treated with pTRV2-*00* or pTRV2-*VdPT1* at 14 and 21 dpi. (**B**) Comparison of vascular browning in the stem segments of HIGS cotton seedlings at 14 dpi. (**C**) Disease index of HIGS cotton seedlings at 14 and 21 dpi. (**D**) qRT-PCR assay of *VdPT1* expression in the root, stem and leaf of HIGS cotton seedlings at 21 dpi. The cotton *tubulin* gene was used as the internal reference gene. (**E**) qRT-PCR assay of fungal biomass in HIGS cotton seedlings at 21 dpi. The data were statistically analyzed used IBM SPSS statistics 26.0. Statistical significance was determined using Student’s *t*-test. ** and * above the error line indicate a significant difference between HIGS cotton seedlings and pTRV2-*00* control at *p* < 0.01 and *p* < 0.05, respectively.
