# Supplementary material for: VdPT1 Encoding a Neutral Trehalase of Verticillium dahliae Is Required for Growth and Virulence of the Pathogen
Source: Int J Mol Sci. 2023 Dec 25;25(1):294. doi: 10.3390/ijms25010294 (PMC10778863; doi:10.3390/ijms25010294)
Supplement: Supplementary file 1 [file ijms-25-00294-s001.zip › Supplement Figure S1-S4.pdf]

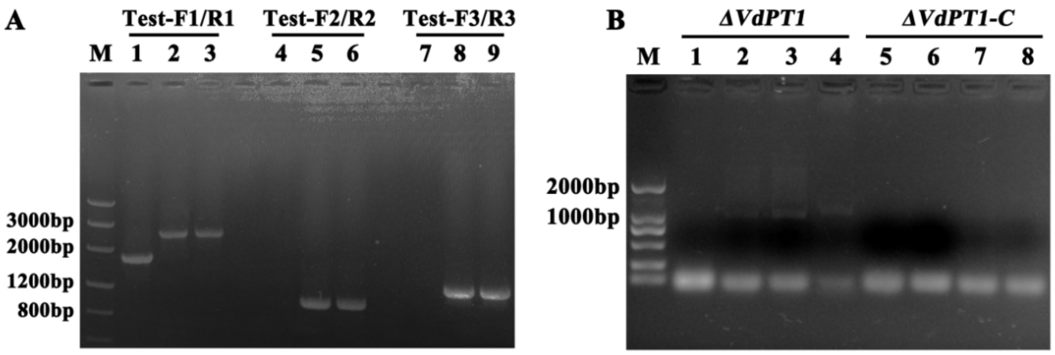

Figure S1. Verification of the *ΔVdPT1* deletion mutants and the *ΔVdPT1-C* complementary strain by PCR.

(A) Lanes 1-3 are PCR amplification results by the primer pair of Test-F1/R1as primes; lanes 4-6 are PCR amplification result by the primer pair of Test-F2/R2; lanes 7-9 are PCR amplification results by the primer pair of Test-F3/R3. M indicates DNA marker. Lanes 2, 3, 5, 6, 8 and 9 are the PCR amplification results of *ΔVdPT1*, and lanes 1, 4 and 7 are the PCR amplification results of wild-type Vd991. (B) PCR validation of the complementary strain *ΔVdPT1-C*. Lanes 2-4 are PCR amplification results of *ΔVdPT1*. Lanes 5-7 are PCR amplification results of the complementary mutant strain *ΔVdPT1-C*. Lanes 1 and 8 are negative controls. M is a DNA ladder (DS2000).

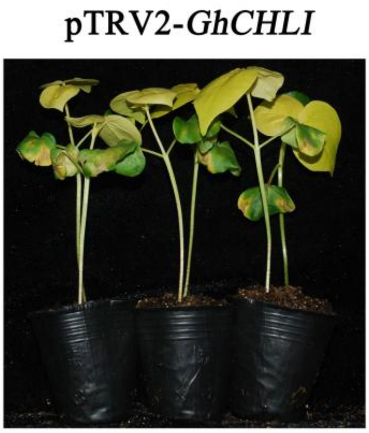

Figure **S2****. Leaf-bleaching phenotype of cotton seedlings treated with pTRV2-*GhCHLI* at 10 days after injection.**

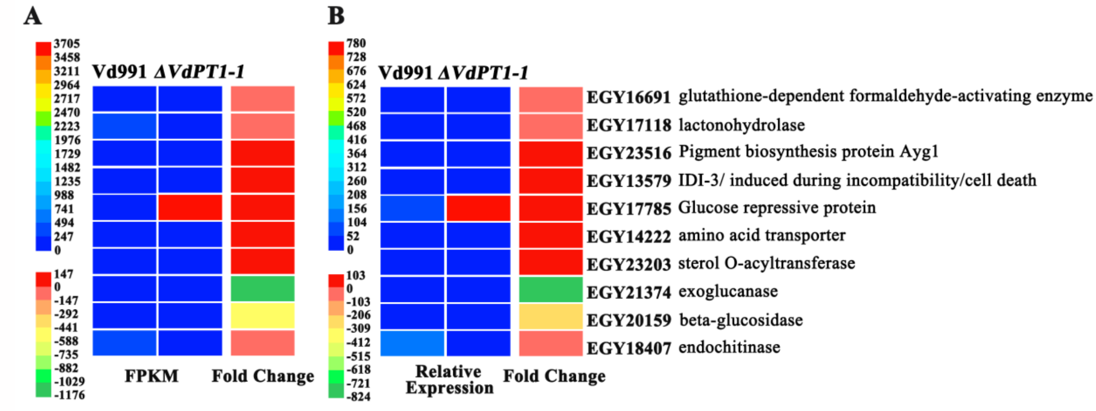

Figure **S3****. Analysis of RNA-seq data reliability.**

(A) Heatmap of the expression level of 10 DEGs based on FPKM values. (B) Heatmap of the expression level of the same 10 DEGs based on qRT-PCR.

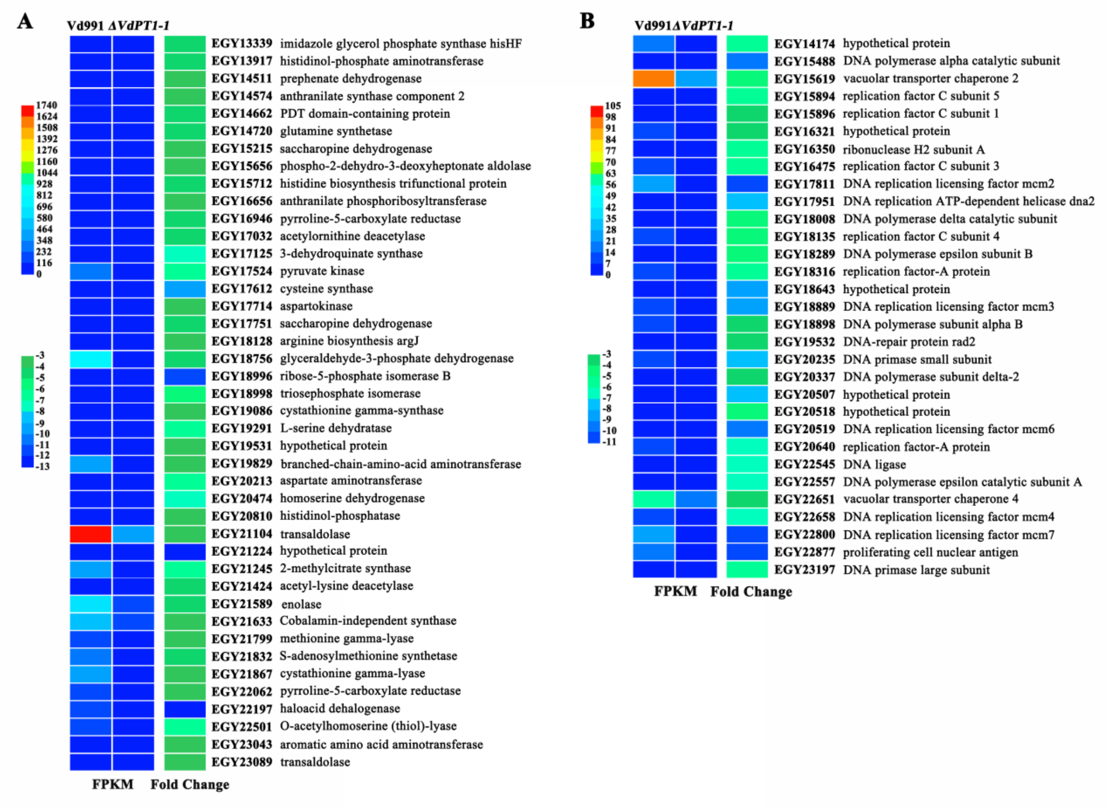

Figure S4. Heatmap of DEGs related to DNA replication and amino acid synthesis.

(A) Heatmap of DEGs related to amino acid synthesis. (B) Heat map of DEGs related to DNA replication.
